# Tolerance, Growth, and Physiological Responses of the Juvenile Razor Clam (*Sinonovacula constricta*) to Environmental Ca^2+^ and Mg^2+^ Concentrations

**DOI:** 10.3389/fphys.2019.00911

**Published:** 2019-07-17

**Authors:** Maoxiao Peng, Zhi Li, Xiaojun Liu, Donghong Niu, Tianyi Lan, Bo Ye, Zhiguo Dong, Jiale Li

**Affiliations:** ^1^Key Laboratory of Exploration and Utilization of Aquatic Genetic Resources, College of Fisheries and Life Science, Shanghai Ocean University, Shanghai, China; ^2^National Demonstration Center for Experimental Fisheries Science Education, Shanghai Ocean University, Shanghai, China; ^3^Co-Innovation Center of Jiangsu Marine Bio-Industry Technology, Jiangsu Ocean University, Lianyungang, China; ^4^Shanghai Engineering Research Center of Aquaculture, Shanghai, China

**Keywords:** *Sinonovacula constricta*, Ca^2+^ and Mg^2+^ ions, survive, growth, physiology

## Abstract

To facilitate transplanting razor clam (*Sinonovacula constricta*) populations to inland saline-alkaline waters (ISWs), we evaluated the tolerance of juvenile *S. constricta* (JSC) to Ca^2+^ and Mg^2+^ concentrations, and determined the effects of these ions on JSC growth and physiological parameters. After 30 days stress, the tolerable ranges of JSC to Ca^2+^ and Mg^2+^ were determined to be 0.19 mmol⋅L^-1^–19.46 mmol⋅L^-1^ and 0 mmol⋅L^-1^–29.54 mmol⋅L^-1^, respectively. The concentrations of Ca^2+^ (less than 0.65 mmol⋅L^-1^ or more than 3.24 mmol⋅L^-1^) and Mg^2+^ (less than 0.37 mmol⋅L^-1^ or more than 14.17 mmol⋅L^-1^) significantly inhibit JSC growth. Physiological enzyme activity no significant response when the concentrations range of Ca^2+^ and Mg^2+^ are 0.93 mmol⋅L^-1^–6.49 mmol⋅L^-1^ and 0.37 mmol⋅L^-1^–14.77 mmol⋅L^-1^, respectively. For transplantation practice, these data indicate that only high concentrations of Ca^2+^ (3.24–6.825 mmol⋅L^-1^) and Mg^2+^ (14.77–33.69 mmol⋅L^-1^) in target inland saline-alkaline water had significantly impact on growth and physiological response. In addition, present study suggests that the increase in Ca^2+^ and Mg^2+^ ion concentrations caused by ocean acidification will not affect the survival, growth and physiology of *S. constricta*. Current research suggests that *S. constricta* can adapt to extreme changes in the marine environment (Ca^2+^ and Mg^2+^) and may be an excellent candidate for inland saline-alkaline water transplantation practice.

## Introduction

Saline-alkali soil and inland saline-alkaline waters (ISWs) exist in more than 100 countries (Shi, 2009). In China, there are approximately 1 million km^2^ of saline-alkaline soil and approximately 0.3 million km^2^ of ISW (He et al., 2010). Saline water resources have long been used for agriculture, but traditional terrestrial crops are not salt-tolerant and cannot be irrigated with salt water. Therefore, ISWs are often low-yielding land (Qadir et al., 2001; Wang et al., 2011). The use of ISWs to culture economically important marine organisms represents an effective means to use inland saline-alkali resources, such as the most successful species *Litopenaeus vannamei* (Roy et al., 2007, 2010). Inland aquaculture of economically important marine organisms worldwide is mainly concentrated in ISW areas, and is practiced in several countries. However, there are few reports on marine mollusc transplanting in ISWs; these reports only describe aquaculture of *Mytilus edulis* (Dinh and Fotedar, 2016), *Ruditapes philippinarum* (Hiele et al., 2014), *Haliotis laevigata* (Doupé et al., 2003), *Trochus niloticus* (Lee, 1997), and *Crassostrea gigas* and *Saccostrea glomerata* (Ingram et al., 2015).

In China, some ISWs have been used for aquaculture, but most of them have historically been in a state of disuse (Lv et al., 2012). According to incomplete statistics, more than 33 economically important organisms are cultured in ISWs in China (mainly freshwater fish) (Zhang and Zhang, 1999). However, the practice of aquaculture of economically important marine mollusc in ISW has only been attempted with *Cyclaina sinensis* (Lin et al., 2012) in China; this venture ultimately did not succeed.

The ion composition of ISW is quite different from that of seawater. Monovalent and divalent cations and their interactions have an important influence on the physiology of organisms (Duchen, 2000). All of the main ionic components in ISW (Ca^2+^, Mg^2+^, Na^+^, K^+^, SO_4_^2-^, HCO_3_^-^, OH^-^, Cl^-^, Ca^2+^: Mg^2+^, Na^+^: K^+^, SO_4_^2-^: Cl^-^) are factors that affect the survival and growth of aquatic animals (Hadfield et al., 2012; Hiele et al., 2014; Raizada et al., 2015). Ca^2+^ is a crucial element of the outer shell of marine crustaceans and mollusc, is involved in muscle activity, shell formation, neurotransmission, and osmotic regulation, and plays an important role in the growth of marine invertebrates such as crustaceans and mollusc (Wilbur and Yonge, 2013; Yang, 2011). Mg^2+^ is found in the active region of many enzymes, it is a metabolic cofactor, and is an important participant in the active transport of substances involved in maintenance of the cell membrane. Studies have shown that Mg^2+^ has an important effect on the survival, growth, and osmotic pressure of marine crustaceans (Mantel and Farmer, 1983). The imbalance of ionic components in ISW tends to affect aquaculture practices more than salinity, as demonstrated in *L. vannamei* by Saoud et al. (2003). In addition, it has been reported that the high content of Mg^2+^, Ca^2+^, and K^+^, and low Ca^2+^/Mg^2+^ and Na^+^/K^+^ ratios in ISW affect the survival of cultured *L. vannamei*.

Many marine invertebrates are osmotic conformers: the osmotic concentration (and usually the ionic composition) of the extracellular fluid is similar to that of the ambient seawater (Larsen et al., 2014). Thus, in aquatic mollusc can be found the stenohaline, euryhaline or oligohaline osmotic conformers, and, also the freshwater bivalves (Deaton, 2008). Most of the intertidal zone bivalve belongs to the euryhaline osmotic conformers, and usually their conformational floor are reported to be between 0.7 ppt and 3.4 ppt (Otto and Pierce, 1981; Deaton, 1992, 2008). There is also volume regulation in marine mollusc, the process that returns the cytoplasmic and extracellular fluid compartments to osmotic equilibrium with the ambient medium (Smith and Pierce, 1987; Deaton, 1992; Larsen et al., 2014). The capacity for volume regulation in marine osmotic conformers varies from limited to high (some molluscs) (Virkar, 1966; Pierce, 1971; Gainey, 1978). Although the study of osmotic and ionic regulation in bivalve is not sufficient, Ca^2+^ and Mg^2+^ play an important role in this system.

The Ca^2+^ and Mg^2+^ ion contents vary widely in ISWs in different regions of China. For example, the Ca^2+^ content of ISWs in Haolebaojinao (Wu Shen banner, Ordos City, the Inner Mongolia Autonomous Region, China) is only 0.199 mmol⋅L^-1^ of seawater (Tian, 2000). In contrast, the Ca^2+^ content of Bosten Lake is 5.61 mmol⋅L^-1^ that of seawater with the same salinity (Shi, 1989). Shi (2009) found that the Mg^2+^ content in ISW in the middle reaches of the Yellow River in China was 2.33–8.15 mmol⋅L^-1^. Therefore, studying the effects of Ca^2+^ and Mg^2+^ on marine invertebrates can help facilitate transplanting marine invertebrates in China ISW.

*Sinonovacula constricta* is a eurythermal and euryhaline marine mollusc that is distributed in estuary and intertidal zones (Lin and Wu, 1990). *S. constricta* feeds on microalgae, and fixes calcium ions in its aquatic environment during the formation of its shell, which can contribute to the improvement of an ISW environment. Peng et al. (2018) reported that *S. constricta* tolerate high pH and carbonate alkalinity under long-term stress, and suggested that *S. constricta* is a potentially good species for culture in ISW. However, the effects of different concentrations of Ca^2+^ and Mg^2+^ on the survival, growth, and physiological responses of *S. constricta* have not been reported. In addition, ocean acidification and climate warming also directly affect the concentration of Ca^2+^ and Mg^2+^ ions in the ocean (Orr et al., 2005). Therefore, understanding the critical tolerances and physiological responses of *S. constricta* to Ca^2+^ and Mg^2+^ stress is crucial for the development of optimal ISW culture conditions and ocean environments changing. Thus, this study investigated the effects of Ca^2+^ and Mg^2+^ on the survival, growth, and physiological responses of juvenile *S. constricta* (JSC), and the possibility of this species as a potential candidate for transplantation to ISWs, providing a theoretical basis for this process.

## Materials and Methods

Experimental animals were treated according to the guidelines of the Institutional Animal Care and Use Committee of Shanghai Ocean University (IACUC-SHOU), Shanghai, China, under examination and approval number SHOU-DW-2018-014. In this tests the animals were artificially propagated as juveniles.

Healthy JSCs (20 days old) were obtained commercially (Zhejiang Sanmen Donghang). Before experimentation, all JSCs were kept in at 8 ppt seawater for 3 days, then were kept at 6 ppt artificial seawater (Ca^2+^: 1.71 mmol⋅L^-1^, Mg^2+^: 9.23 mmol⋅L^-1^) for 4 days. In these conditions the total salinity of water is set at 6 ppt, since the salinity of ISW (carbonate type) in China is around 6 ppt (Yao et al., 2010). Artificial seawater was formulated using chemical reagents (AR degree, Sangon Biotech) and distilled water according to the Artificial Seawater Recipe (Chu) in Table 1A (Harvey, 1957; Yao et al., 2010). Approximately 10,000 specimens of JSCs were obtained before experiments, and then 270 JSCs were randomly selected to measure shell length (0.2102 ± 0.058 cm) and body weight (0.000892 ± 0.00015 g). Water temperature in experiments was maintained between 20 to 22°C.

**Table 1A:** Artificial seawater recipe (Chu).

Reagents	Dosage (mg⋅L^-1^)	Dosage (mmol⋅L^-1^)
NaCl	4,143	70.89322382
MgCl_2_	879	9.232223506
Na_2_SO_4_	691.2352941	4.866483344
CaCl_2_	190.0305882	1.712303012
KCl	127.7647059	1.713812285
NaHCO_3_	33.88235294	0.403313331
KBr	16.94117647	0.142360435
H_2_BO_3_	4.588235294	0.075451986
Al_2_(SO_4_)_3_	0.529411765	0.001547336
BaCl_2_.2H_2_O	0.015882353	6.50303E-05
LiNO_3_	0.176470588	0.002559771
SrCl_2_	4.235294118	0.026717727
NaF	0.529411765	0.012605642
NaNO_3_	8.823529412	0.103806228
Na_2_HPO_4_	0.882352941	0.006214891
Na_2_SiO_3_.9H_2_O	1.764705882	0.006211894
MnCl_2_	0.035294118	0.000280473
FeC_6_H_5_O_7_.3H_2_O	0.095294118	0.000318875
CuSO_4_.5H_2_O	0.007058824	2.82799E-05
ZuSO_4_.7H_2_O	0.001764706	6.13939E-06
(NH_4_)_6_Mo_7_O_24_.4H_2_O	0.004411765	3.5702E-06

### Long-Term Stress Study

Juvenile *S. constrictas* were divided into 29 groups for long-term stress testing (Table 1B), consisting of a control group (CK), 14 groups with different concentrations of Mg^2+^ (DCMg: G1-G14), and 14 groups with different concentrations of Ca^2+^ (DCCa: G1–G14). Each of the 29 groups had 3 parallel trials. For each parallel stress trial, 90 JSCs were cultured in an 11 cm diameter round plate for a month. 150 mL test water (with the appropriate salinity and Ca^2+^ or Mg^2+^ concentration) was added to the round plate, and the test water was changed once every single day. Feed-water was prepared as follows: test water was used to replace the culture water of *Chaetoceros calcitrans* by centrifugation, and the concentration of *C. calcitrans* was adjusted to 400–480 cells μL^-1^ in test water. For every single feeding, 15 mL feed-water was used to replace 15 mL of test water. JSCs were fed thrice a day.

**Table 1B:** Design of different Ca^2+^ and Mg^2+^ concentrations for the long-term stress test.

Treatments	CK^ab^	DCMg-G1^b^	DCMg-G2	DCMg-G3	DCMg-G4	DCMg-G5	DCMg-G6	DCMg-G7	DCMg-G8	DCMg-G9	DCMg-G10^b^	DCMg-G11	DCMg-G12	DCMg-G13	DCMg-G14
Ca^2+^ (mmol⋅L^-1^)	1.71	1.71	1.71	1.71	1.71	1.71	1.71	1.71	1.71	1.71	1.71	1.71	1.71	1.71	1.71
Mg^2+^ (mmol⋅L^-1^)	9.23	0	0.046	0.092	0.18	0.37	1.85	3.69	14.77	29.54	73.86	147.72	211.02	295.43	379.84
Ca^2+^/Mg^2+^ ratio	0.19	–	37.13	18.57	9.28	4.64	0.93	0.46	0.12	0.058	0.023	0.012	0.0081	0.0058	0.0035
Treatments	–	DCCa-G1^b^	DCCa-G2	DCCa-G3	DCCa-G4	DCCa-G5	DCCa-G6	DCCa-G7	DCCa-G8	DCCa-G9	DCCa-G10	DCCa-G11	DCCa-G12	DCCa-G13^b^	DCCa-G14
Mg^2+^ (mmol⋅L^-1^)	–	9.23	9.23	9.23	9.23	9.23	9.23	9.23	9.23	9.23	9.23	9.23	9.23	9.23	9.23
Ca^2+^ (mmol⋅L^-1^)	–	0	0.093	0.19	0.28	0.65	0.93	2.32	3.24	6.49	19.46	51.89	103.78	207.55	415.1
Ca^2+^/Mg^2+^ ratio	–	–	0.01	0.02	0.03	0.07	0.1	0.25	0.35	0.7	2.11	5.62	11.24	22.48	44.96

*^*a*^Only one number of control group (CK) be set in long-term stress study. ^*b*^The groups were used in acute stress study.*

Live JSCs were counted at day 0 (0-day) and at day 30 (30-day). At 0-day, 36 JSCs (6 repeats: 6 JSCs per repeat) were selected to determine the activities of the following enzymes: Na^+^/K^+^-ATPase (NKA), aspartate aminotransferase (AST), superoxide dismutase (SOD), acetylcholinesterase (AChE), and lysozyme (LZM). At 30-day, live JSCs were used to measure shell length, body weight, and enzymes activity (NKA, SOD, AST, AChE, and LZM). Nine JSCs (representing three separate tests with three JSCs in each group) were used to measure enzyme activities. Briefly, after shell removal, JSC tissue was homogenized in normal saline water in a ratio of 1 mg: 9 μl. After homogenization, the total protein content of the sample was measured by Coomassie Brilliant Blue Total Protein Assay kit (Nanjing Jiancheng Bioengineering Institute, China), according to the manufacturer’s instructions. NKA, AST, SOD, AChE, and LZM enzyme activities were measured by corresponding kits from Nanjing Jiancheng Bioengineering Institute Ltd., as previously described (Peng et al., 2018).

Na^+^/K^+^-ATPase activity was measured by Pi released from ATP, in the incubation solution containing: 100 mM NaCl, 20 mM KCl, 4 mM MgCl_2_, 1 mM EGTA, 0.2 mM 2-methyl-8-(phenylmethoxy)imidazol(1,2-α) pyridine-3-acetonitrile 40 mM Tris–HCl (pH 7.4) and 3 mM Na_2_ATP. The incubation time with substrate was 10 min. Pi concentration was calculated from the regression line based on standard Pi solutions. The units of NKA activity are expressed as U (μmol Pi mg prot^-1^ h^-1^). AST can transfer amino and keto groups between alpha ketoglutarate and aspartic acid, then generate glutamic acid, and oxaloacetate. Oxaloacetate can be decarboxylated to pyruvic acid by itself during the reaction. Pyruvic acid reacts with 2,4-Dinitrophenylhydrazine to form 2,4-Dinitrophenylhydrazone, which is reddish brown in an alkaline solution. After measuring the OD, the standard curve was checked to obtain AST activity, which is expressed as U (μmol Pyruvic acid mg prot^-1^ min^-1^). SOD activity was tested using SOD assay kit by WST-1 method. The WST-1 method depends on produces a water-soluble formazan dye upon reduction with the superoxide anion. The rate of the reduction of WST-1 with O2^-^ are linearly related to the xanthine oxidase activity, and this reduction is inhibited by SOD. Therefore, the 50% inhibition activity of SOD can be determined by the colorimetric method. The SOD activity is expressed as units per mg of protein (U mg prot^-1^), where the one U represents the amount of enzyme required to achieve a SOD inhibition rate of 50%. AChE hydrolyzes acetylcholine to produce choline and acetic acid. Choline can react with sulfhydryl chromogenic reagent to form Sym-Trinitrobenzene (yellow compound), after measuring the OD, the standard curve was checked to obtain AChE activity, which is expressed as units per mg of protein (U mg prot^-1^), where the one U represents at 37°C for 6 min of reaction conditions, the amount of enzyme required to hydrolyze 1 mmol of acetylcholine. The activity of LZM was detected using a turbidimetric method, which light transmittance of the bacterial turbid solution. Lysozyme can lysis bacteria by hydrolyze peptidoglycan on the bacterial wall to increase the light transmission of the bacterial turbid solution. After measuring the light transmission, the standard curve was checked to obtain LZM activity, which is expressed as units per mg of protein (U mg prot^-1^), where the one U equivalent to the activity capacity of standard LZM. All data of enzyme activities are ultimately represented as a ratio of 30-day to 0-day.

### Acute Stress Study

Within 3 days of the start of the long-term stress study, we found that all JSCs died in the following groups: DCMg-G10, DCMg-G11, DCMg-G12, DCMg-G13, DCMg-G14, DCCa-G13, and DCCa-G14. To more fully explain the physiological effects of Ca^2+^ and Mg^2+^ on JSCs, we designed and conducted an acute stress study. The CK, DCMg-G1, DCMg-G10, DCCa-G1, and DCCa-G13 were used in the acute stress experiment. In this experiment, the animals were exposed to the same conditions used in the long-term stress tests, except that the clams were not fed algae. At 0-h, 12-h, 24-h, and 48-h, ten JSCs from each group were used to measure oxygen consumption, then the hemolymph of JSCs was collected, as previously reported (Peng et al., 2017), to separate haemocytes and to measure metabolic and phagocytic activities.

The method used for measuring oxygen consumption rate was previously reported (Peng et al., 2019). After hemolymph collection, 20 μL of JSC haemocyte suspension solution was diluted with TBS to 100 μL. Then 10 μL of 1/10 diluted carboxylate-modified microspheres (diameter 1 μm, yellow-green fluorescent, FluoSpheres^®^, Invitrogen) was added, and samples were incubated in the dark at room temperature for 1 h (Wang et al., 2014). A BD C6Plus (BD Biosciences, United States) flow cytometer was used to measure the phagocytosis of haemocytes. The same haemocyte concentration without fluorescent microspheres in each group was analyzed by flow cytometry to obtain the cell concentration, and we set the threshold to be Gate A (cell only). The relative size of haemocytes (FSC value) and FITC fluorescence intensity (FITC value) were selected to limit the data. Finally, the data was extracted for analysis and figure mapping.

The metabolic activity of haemocytes was measured with a Cell Counting Kit-8 (CCK-8, Dojindo, Japan) assay. In every parallel group, 20 μL haemocyte suspension solution was diluted with TBS to 200 μL. Then, 20 μL of CCK-8 reagent was added and incubated at 28°C for 3 h. For the CK, 20 μL serum was obtained from the haemocyte suspension solution, and was diluted with TBS to 200 μL; then 0 μL of CCK-8 was added and incubated at 28°C for 3 h. After the OD value was measured, the relative metabolic activity was calculated using OD value and the total number of haemocytes (obtained from the phagocytosis test).

### Calculation and Data Analytical Methods

Survival rate (SR):

$$\begin{array}{c} SR(\%)\;=\;100\;\times \;N_{30}/N_{0} \end{array}$$

*N_30_* and *N_0_* are the numbers of surviving individuals in the 30-day and 0-day groups, respectively.

Shell length growth rate (SGR):

$$\begin{array}{c} SGR(\%day^{-1})\;=\;100\;\times \;(L_{30}\;-\;L_{0})/T, \end{array}$$

*L_30_* and *L_0_* are the average lengths of the individuals in the 30-day and 0-day groups, respectively. *T* is 30 days.

Weight gain rate (WG):

$$\begin{array}{c} WG(\%day^{-1})\;=\;100\;\times \;(W_{30}\;-\;W_{0})/T \end{array}$$

*W_30_* and *W_0_* are the average wet body weights of the individuals in the 30-day and 0-day groups, respectively. *T* is 30 days.

Oxygen consumption rate:

$$\begin{array}{c} (mgO_{2}\cdot g^{-1}\cdot L^{-1}\cdot h^{-1})\;=\;[A_{i}(initialoxygenconcentrations)\;-A_{f}(finaloxygenconcentrations)]\;\times \;V\;\\ \left(volumeoftestwater)\;/\;[W(wetbodyweight)\;\times \;T(testtime)\right] \end{array}$$

Phagocytosis rate and relative metabolic activity:

$$\begin{array}{c} Phagocytosisrate\%\;=\;(H_{t}\;-\;H_{u})/H_{t}\;\times \;100 \end{array}$$

$$\begin{array}{c} Relativemetabolicactivity\;=\;[(OD_{G}\;-\;OD_{\mathrm{GB}})/H_{\mathrm{tG}}\;\times \;1000]/[(OD_{\mathrm{CK}}\;-\;OD_{\mathrm{CKB}})/H_{\mathrm{tCK}}\;\times \;1000] \end{array}$$

H_t_ and H_u_ are the total haemocyte and un-phagocytic haemocyte numbers, respectively. OD_G_, OD_GB_, and H_tG_ are the OD of test sample, the blank OD of test sample, and the total haemocyte counts of the test sample, respectively. OD_CK_, OD_CKB_, and H_tCK_ are the OD of control sample, the blank OD of control sample and the total haemocyte count of the control sample, respectively.

### Statistical Analysis

Sigmaplot 12.3 software was used to identify significant differences analysis and to plot results. Significant differences were analyzed by using single factor analysis of variance (one-way ANOVA analysis) and the LSD method.

## Results

### Survival and Growth of JSC Under Long-Term Ca^2+^ or Mg^2+^ Stress

**FIGURE 1:**
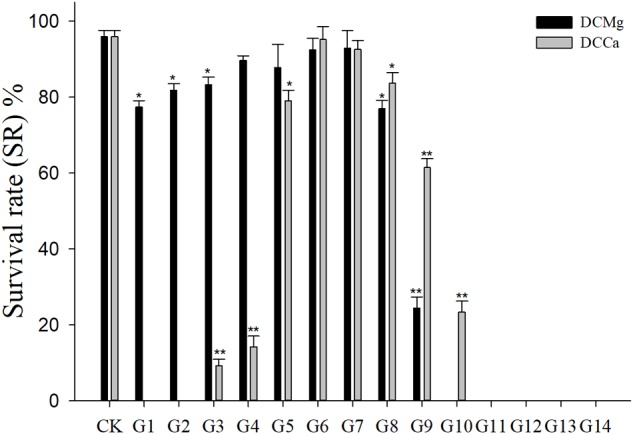
Survival rates of JSCs under long-term stress with different Ca^2+^ and Mg^2+^ concentrations. Bars (mean ± SE, *n* = 3) with an asterisk denote a significant difference (^∗^*P* < 0.05, ^∗∗^*P* < 0.001) between the test groups (G1–G14) and control group (CK).

During the long-term stress, we found that the concentrations of Mg^2+^ (higher than 73.86 mmol L^-1^) and Ca^2+^ (higher than 207.55 mmol L^-1^) cause JSCs to die rapidly (all JSC death groups are listed in Table 2). The concentrations of Ca^2+^ (lower than 0.093 mmol L^-1^) causes JSCs to die over an extended period of time. Meanwhile, we observed that the bodies of JSCs were lacked calcium carbonate shells in groups stressed with Ca^2+^ concentrations lower than 0.093 mmol L^-1^. However, the JSCs stressed with low concentrations of Mg^2+^ remained viable (Figure 1). In the range of Mg^2+^ concentration from 0 mmol L^-1^ to 29.54 mmol L^-1^, there is a trend of JSC survival ratio show as first rise and then fall. Similarly, in the range of Ca^2+^ concentration from 0.19 mmol L^-1^ to19.46 mmol L^-1^, there is a trend for JSC survival to first rise and then fall. The Mg^2+^ concentration from 0.18 mmol L^-1^ to 3.69 mmol L^-1^ and Ca^2+^ concentration from 0.93 mmol L^-1^ to 2.32 mmol L^-1^ showed no significant differences in survival compared to the CK.

**Table 2:** Detailed juvenile *Sinonovacula constricta* mortality information in the test groups which cannot survive under long-term stress with different Ca^2+^ and Mg^2+^ concentrations.

Time	Number of death events										
	DCCa-G1	DCCa-G2	DCCa-G11	DCCa-G12	DCCa-G13	DCCa-G14	DCMg-G10	DCMg-G11	DCMg-G12	DCMg-G13	DCMg-G14
1-day	0.33 ± 0.58	0.00 ± 0.00	0.67 ± 0.58	0.67 ± 0.58	57.33 ± 4.04	90 ± 0.00	18.33 ± 4.04	90 ± 0.00	90 ± 0.00	90 ± 0.00	90 ± 0.00
2-day	0.00 ± 0.00	0.67 ± 0.58	1.00 ± 1.00	8.33 ± 1.16	24.33 ± 2.52	–	55.67 ± 4.62	–	–	–	–
3-day	1.00 ± 1.00	0.33 ± 0.58	3.33 ± 1.53	17.67 ± 2.52	8.33 ± 1.53	–	16.00 ± 1.73	–	–	–	–
4-day	0.33 ± 0.58	0.33 ± 0.58	31.00 ± 2.65	48.67 ± 4.04	–	–	–	–	–	–	–
5-day	2.00 ± 2.00	0.67 ± 1.16	18.67 ± 1.53	14.67 ± 3.06	–	–	–	–	–	–	–
6-day	1.33 ± 0.58	0.67 ± 0.58	8.33 ± 1.53	–	–	–	–	–	–	–	–
7-day	1.67 ± 1.53	2.00 ± 1.00	4.33 ± 1.16	–	–	–	–	–	–	–	–
8-day	0.67 ± 1.16	1.33 ± 1.16	7.00 ± 1.00	–	–	–	–	–	–	–	–
9-day	1.67 ± 1.53	0.67 ± 1.16	4.67 ± 1.53	–	–	–	–	–	–	–	–
10-day	1.67 ± 2.08	1.67 ± 1.16	4.00 ± 1.00	–	–	–	–	–	–	–	–
11-day	0.67 ± 1.16	0.67 ± 0.58	3.00 ± 1.00	–	–	–	–	–	–	–	–
12-day	1.00 ± 1.00	0.67 ± 1.16	2.33 ± 0.58	–	–	–	–	–	–	–	–
13-day	8.00 ± 2.00	0.33 ± 0.58	0.67 ± 0.58	–	–	–	–	–	–	–	–
14-day	8.00 ± 1.00	4.67 ± 2.52	1.00 ± 1.00	–	–	–	–	–	–	–	–
15-day	10.33 ± 6.66	4.33 ± 3.22	–	–	–	–	–	–	–	–	–
16-day	33.33 ± 7.02	6.33 ± 1.53	–	–	–	–	–	–	–	–	–
17-day	4.67 ± 4.16	7.67 ± 1.53	–	–	–	–	–	–	–	–	–
18-day	7.33 ± 2.31	19.33 ± 2.52	–	–	–	–	–	–	–	–	–
19-day	2.33 ± 0.58	8.33 ± 2.08	–	–	–	–	–	–	–	–	–
20-day	3.67 ± 4.04	7.67 ± 1.53	–	–	–	–	–	–	–	–	–
21-day	–	9.33 ± 1.16	–	–	–	–	–	–	–	–	–
22-day	–	11.00 ± 4.58	–	–	–	–	–	–	–	–	–
23-day	–	1.33 ± 1.53	–	–	–	–	–	–	–	–	–

*^∗^Data present in this table as mean ± standard error.*

**FIGURE 2:**
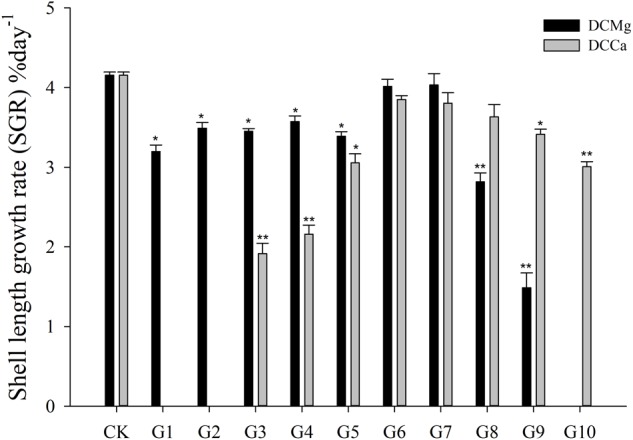
Daily shell length growth rate of JSCs under long-term stress with different Ca^2+^ and Mg^2+^ concentrations. Bars (mean ± SE, *n* = 3) with an asterisk denote a significant difference (^∗^*P* < 0.05, ^∗∗^*P* < 0.001) between the test groups (G1–G10) and CK.

**FIGURE 3:**
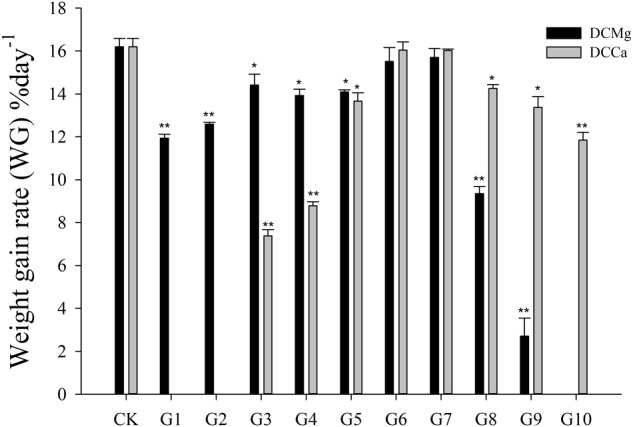
Daily weight gain rate of JSCs under long-term stress with different Ca^2+^ and Mg^2+^ concentrations. Bars (mean ± SE, *n* = 3) with an asterisk denote a significant difference (^∗^*P* < 0.05, ^∗∗^*P* < 0.001) between the test groups (G1–G10) and CK.

The shell growth rate was significantly inhibited in the Mg^2+^ concentration from 0 mmol L^-1^ to 0.37 mmol L^-1^ (*P* < 0.05), and from 14.77 mmol L^-1^ to 29.54 mmol L^-1^ (*P* < 0.001), and was significantly inhibited in the Ca^2+^ concentration 0.19–0.65 mmol⋅L^-1^ (*P* < 0.05) and 6.49–19.46 mmol⋅L^-1^ (*P* < 0.05) (Figure 2). The body weight gain rate was significantly inhibited in the Mg^2+^ concentration 0–0.37 mmol⋅L^-1^ (*P* < 0.05) and 14.77–29.54 mmol⋅L^-1^ (*P* < 0.001), and was significantly inhibited in the Ca^2+^ concentration 0.19–0.65 mmol⋅L^-1^ (*P* < 0.05) and 3.24–19.46 mmol⋅L^-1^ (*P* < 0.05) (Figure 3).

### Enzyme Activity of JSC Under Long-Term Ca^2+^ or Mg^2+^ Stress

**FIGURE 4:**
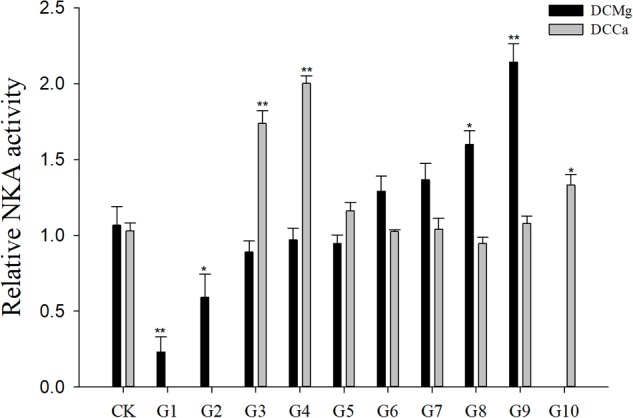
Relative NKA activity of JSCs under long-term stress with different Ca^2+^ and Mg^2+^ concentrations. Results show NKA activity at 30-days normalized to the 0-day. Bars (mean ± SE, *n* = 3) with an asterisk denote a significant difference (^∗^*P* < 0.05, ^∗∗^*P* < 0.001) between the test groups (G1–G10) and CK.

**FIGURE 5:**
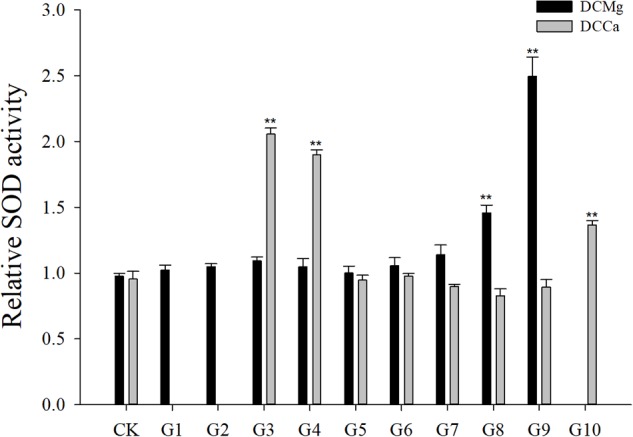
Relative SOD activity of JSCs under long-term stress with different Ca^2+^ and Mg^2+^ concentrations. Results show SOD activity at 30-days normalized to the 0-day. Bars (mean ± SE, *n* = 3) with an asterisk denote a significant difference (^∗^*P* < 0.05, ^∗∗^*P* < 0.001) between the test groups (G1–G10) and CK.

**FIGURE 6:**
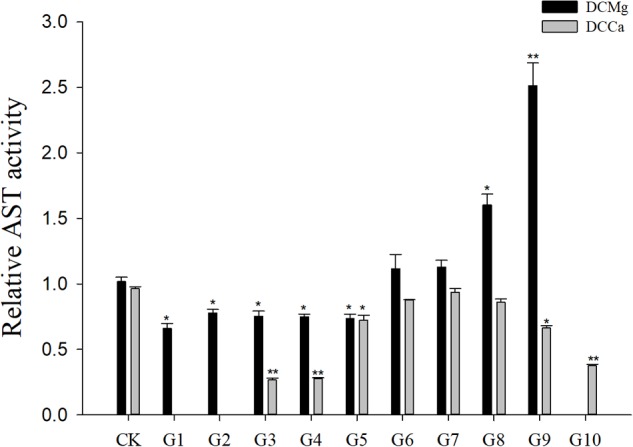
Relative AST activity of JSCs under long-term stress with different Ca^2+^ and Mg^2+^ concentrations. Results show AST activity at 30-days normalized to the 0-day. Bars (mean ± SE, *n* = 3) with an asterisk denote a significant difference (^∗^*P* < 0.05, ^∗∗^*P* < 0.001) between the test groups (G1–G10) and CK.

**FIGURE 7:**
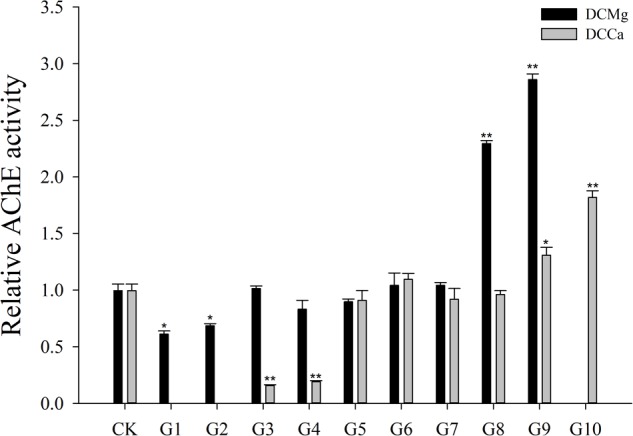
Relative AChE activity of JSCs under long-term stress with different Ca^2+^ and Mg^2+^ concentrations. Results show AChE activity at 30-days normalized to the 0-day. Bars (mean ± SE, *n* = 3) with an asterisk denote a significant difference (^∗^*P* < 0.05, ^∗∗^*P* < 0.001) between the test groups (G1–G10) and CK.

**FIGURE 8:**
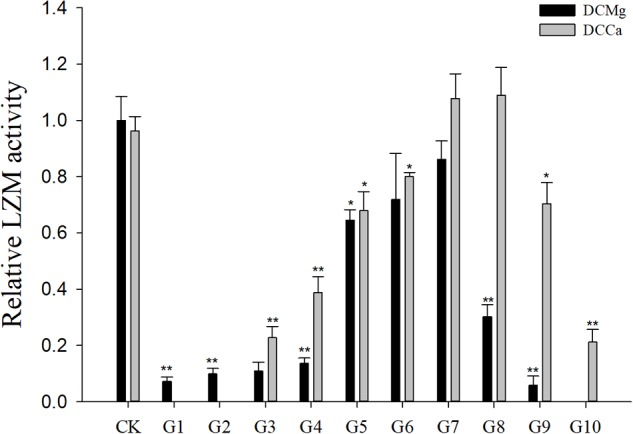
Relative LZM activity of JSCs under long-term stress with different Ca^2+^ and Mg^2+^ concentrations. Results show LZM activity at 30-days normalized to the 0-day. Bars (mean ± SE, *n* = 3) with an asterisk denote a significant difference (^∗^*P* < 0.05, ^∗∗^*P* < 0.001) between the test groups (G1–G10) and CK.

Na^+^/K^+^-ATPase activity was significantly decreased in JSCs stressed with low concentrations range (0–0.046 mmol⋅L^-1^) of Mg^2+^ (Figure 4). However, 14.77 mmol⋅L^-1^ of Mg^2+^ significantly increased NKA activity in JSCs (*P* < 0.05). Low concentrations and high concentrations of Ca^2+^ affected JSC NKA activity. NKA activity was increased significantly in the Ca^2+^ concentration of 0.19 mmol⋅L^-1^ (*P* < 0.001), 0.28 mmol⋅L^-1^ (*P* < 0.001), and 19.46 mmol⋅L^-1^ (*P* < 0.05). NKA and SOD activity patterns are totally consistent when Ca^2+^ varying. In contrast with NKA, SOD activity is not affected by low Mg^2+^ (Figure 5). AST activity (Figure 6) was significantly reduced in low concentrations (0–0.37 mmol⋅L^-1^) of Mg^2+^ (*P* < 0.05), but was significantly increased in the high Mg^2+^ concentration, 14.77 mmol⋅L^-1^ (*P* < 0.05) and 29.54 mmol⋅L^-1^ (*P* < 0.001). However, both 0.19–0.65 mmol⋅L^-1^ (*P* < 0.05) and 6.49–19.46 mmol⋅L^-1^ (*P* < 0.05) concentrations of Ca^2+^ significantly reduced AST activity in JSCs. AChE activity increased as the concentration of Mg^2+^ increased (Figure 7). AChE activity was inhibited significantly in the Mg^2+^ concentration of 0 mmol⋅L^-1^ and 0.046 mmol⋅L^-1^ (*P* < 0.05), but was increased significantly in the of 14.77 mmol⋅L^-1^ and 29.54 mmol⋅L^-1^ (*P* < 0.001). The same trend was observed in the effects of Ca^2+^ concentration on AChE activity. AChE activity was inhibited significantly in the Ca^2+^ concentration of 0.19 mmol⋅L^-1^ and 0.28 mmol⋅L^-1^ (*P* < 0.001), but was increased significantly in the 6.49 mmol⋅L^-1^ and 19.46 mmol⋅L^-1^ (*P* < 0.001). Only the Mg^2+^ – 1.85 mmol⋅L^-1^, Mg^2+^ – 3.69 mmol⋅L^-1^, Ca^2+^ – 2.32 mmol⋅L^-1^, and Ca^2+^ – 3.24 mmol⋅L^-1^ showed no significant inhibition of LZM activity in JSCs (Figure 8).

### Oxygen Consumption Rate, Phagocytic Rate, and Metabolism Activity of JSC Under Acute Ca^2+^ or Mg^2+^ Stress

**FIGURE 9:**
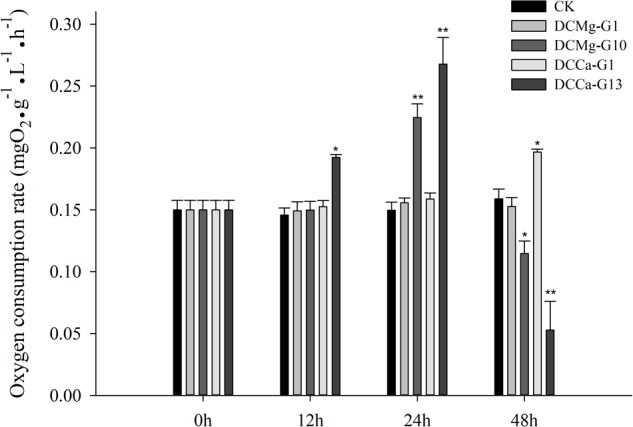
Acute oxygen consumption rate of JSCs under extreme Ca^2+^ and Mg^2+^ concentrations. Bars (mean ± SE, *n* = 3) with an asterisk denote a significant difference (^∗^*P* < 0.05, ^∗∗^*P* < 0.001) between the test groups and CK.

**FIGURE 10:**
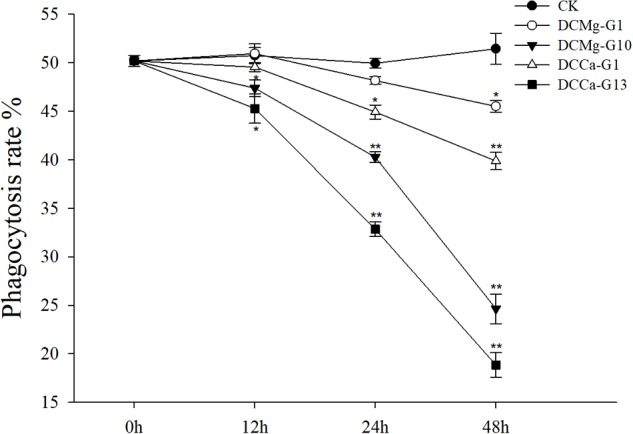
Acute phagocytosis rate of JSCs under extreme Ca^2+^ and Mg^2+^ concentrations. Bars (mean ± SE, *n* = 3) with an asterisk denote a significant difference (^∗^*P* < 0.05, ^∗∗^*P* < 0.001) between the test groups and CK.

**FIGURE 11:**
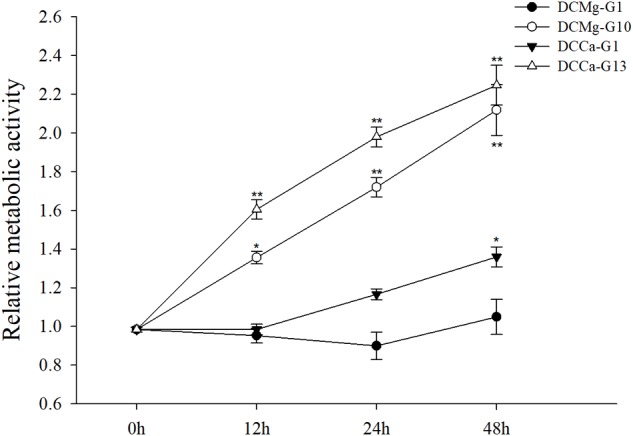
Acute metabolism of JSCs under extreme Ca^2+^ and Mg^2+^ concentrations. Results show metabolism activity of test groups normalized to the 0 h group. Bars (mean ± SE, *n* = 3) with an asterisk denote a significant difference (^∗^*P* < 0.05, ^∗∗^*P* < 0.001) between the test groups and CK.

The oxygen consumption rate of JSCs changed under acute stress due to different concentrations of Ca^2+^ and Mg^2+^ (Figure 9). Within 48 h, the oxygen consumption rate at 0 mmol⋅L^-1^ of Mg^2+^ did not show any significant difference. However, the oxygen consumption rate in the Mg^2+^ concentration 73.86 mmol⋅L^-1^ was significantly increased at 24 h, and then was significantly decreased at 48 h. The oxygen consumption rate of the Ca^2+^ concentration 0 mmol⋅L^-1^ was significantly increased at 48 h. The oxygen consumption rate of the Ca^2+^ concentration 207.55 mmol⋅L^-1^ was significantly increased at 12 and 24 h, and then was significantly decreased at 48 h. The phagocytosis rate of all groups decreased over time (Figure 10). Among them, the Mg^2+^ concentration 0 mmol⋅L^-1^ showed a significant decreased at 48 h (*P* < 0.05), the Mg^2+^ concentration 73.86 mmol⋅L^-1^ showed a decrease at 24 h (*P* < 0.05) and 48 h (*P* < 0.001), and the Ca^2+^ concentration 0 mmol⋅L^-1^ and 207.55 mmol⋅L^-1^ began to decrease significantly at 12 h. The Mg^2+^ concentration 0 mmol⋅L^-1^ showed no significant change in metabolic activity (Figure 11). Metabolic activity was only significantly increased at 48 h in the Ca^2+^ concentration 0 mmol⋅L^-1^. However, metabolic activity of the Mg^2+^ concentration 73.86 mmol⋅L^-1^ and Ca^2+^ concentration 207.55 mmol⋅L^-1^ began to increase significantly at 12 h.

## Discussion

Changes of Ca^2+^ and Mg^2+^ content in water not only affect the survival and growth of bivalves, but also affect their physiological responses. ISW in northwest China, especially the Gansu area (a target area for the transplantation of *S. constricta*), is characterized by broad variation in pH, carbonate alkalinity, and Ca^2+^ (0.775 mmol⋅L^-1^–6.825 mmol⋅L^-1^) and Mg^2+^ (3.07 mmol⋅L^-1^–33.69 mmol⋅L^-1^) content. Studying the ion tolerance range of *S. constricta* is the first and crucial step toward transplanting *S. constricta* cultures to ISW.

There is a significant difference in the tolerance of Ca^2+^ and Mg^2+^ between different invertebrate species. Zhou et al. (2007) reported that the optimal concentrations of Ca^2+^ and Mg^2+^ for the survival of *L. vannamei* larvae were 0.76–8.78 mmol⋅L^-1^ and 0.36–35.51 mmol⋅L^-1^, respectively. Roy et al. (2007) found that Mg^2+^ concentrations are very important for shrimp. The concentration of Mg^2+^ in inland saline-alkali water is at least 25% higher than that of seawater at the same salinity. Sprung (1987) reported that if environmental Ca^2+^ concentrations are less than 0.3 mmol⋅L^-1^, the veliger larvae of the zebra mussel (*Dreissena polymorpha*) cannot survive. Furthermore, the New Zealand mud snail (*Potamopyrgus antipodarum*) is reported to have a minimum Ca^2+^ concentration tolerance of 0.125 mmol⋅L^-1^ (Herbst et al., 2008). Lin et al. (2012) studied the effects of acute Ca^2+^ and Mg^2+^ concentrations on the survival of *C. sinensis* to assess the possibility of cultivating *C. sinensis* as a cultured species in ISW. It was found that when Ca^2+^ concentrations were between 0.11 mmol⋅L^-1^ and 115 mmol⋅L^-1^, and Mg^2+^ concentrations were between 0 mmol⋅L^-1^ and 126.67 mmol⋅L^-1^, the *C. sinensis* survival rates were 66 and 91.5%, respectively. It was determined that Mg^2+^ concentration may not be a primary limiting factor for transplanting *C. sinensis* in ISW. Our findings are similar to those of Lin et al. (2012) namely that JSC can maintain a high survival rate for at least a month when under Mg^2+^ (0 mmol L^-1^) stress. However, the tolerance of JSC to high concentrations of Mg^2+^ is far less than that of *C. sinensis*. It is worth noting that the maximum tolerated concentration of JSC for Mg^2+^ is also slightly lower than the maximum measured value of the target-ISW. This suggests that perhaps Mg^2+^ be one of the limiting factors to the *S. constricta* transplantation. Thus special attention have to be paid to whether the concentration of Mg^2+^ ions too high when practicing transplantation. Mg^2+^-free medium have little effect on the survival rates of JSC and *C. sinensis*; this may be caused by the presence of dietary Mg^2+^ ions, or may indicate that the effect of free Mg^2+^ on survival is a long-term process. Because Dietz et al. (1994) have reported that *D. polymorpha* depleted of Mg^2+^ did not survive beyond 51 days. JSC can survival in a wide range of concentrations of Ca^2+^ (0.19–19.46 mmol⋅L^-1^), suggesting that transplantation of JSC to target-ISW may without effect of Ca^2+^, and could be performed in a broad range of Ca^2+^ concentrations.

The shell of bivalves serves as the first barrier to resist external threats, and the main component of the shell is calcium carbonate. The growth of shells is directly related to the growth of the bivalve. Since Ca^2+^ is the main cation involved in the shell formation of bivalves, it was chosen in previous growth models (Hincks and Mackie, 1997). Most of the environmental calcium (approximately 80%) that is actively taken up from the water is deposited in the shell (Van Der Borght and Van Puymbroeck, 1966). However, these reactions are reversible, and under condition of low Ca^2+^ concentrations, calcium is removed from the shell (Van Der Borght and Van Puymbroeck, 1966). Accordingly, in this test we observed that JSCs died from calcium carbonate shell loss when Ca^2+^ was less than 0.19 mmol⋅L^-1^. Such a situation may stem from the results of Ca^2+^ regulation. On the one hand, it is the release of Ca^2+^ in the shell caused by the efflux of free Ca^2+^ from the hemolymph and shell-mantle fluid. On the other hand, the efflux of Ca^2+^ also causes an increase in free amino acids and acidic metabolites in the body. These acidic end products are buffered by the mobilization of Ca^2+^ from the shell (Akberali et al., 1977). In addition, Wu et al. (2003) studied the effect of Ca^2+^ on the activity of digestive enzymes in *S. constricta*. It was found that an imbalance of Ca^2+^ concentration significantly decreased the activities of amylase and protease, thereby inhibiting growth. The higher Ca^2+^ concentration range of target-ISW 6.49–6.825 mmol⋅L^-1^ and 3.24–6.825 mmol⋅L^-1^ may significantly inhibit the shell length and body weight growth of JSC, respectively. This suggest that transplanting *S. constricta* in the target-ISW may not have growth inhibition caused by Ca^2+^ deficiency, and there may be only a physiological response by excessive Ca^2+^. Other hand suggest that the effect of Ca^2+^ on the growth of JSC may be more than one aspect, which weight is more likely to be affected than shell.

Mg^2+^ is the most abundant divalent ion within living cells. It is a cofactor in many enzymes, and its free ionic concentration regulates metabolic and shell formation processes (Bentov and Erez, 2006; Roy et al., 2010; Marin et al., 2012; Suzuki and Nagasawa, 2013; Wilbur and Yonge, 2013; González-Vera and Brown, 2017). However, Wu et al. (2003) found that the concentration of Mg^2+^ in the water did not affect the digestive enzyme activity of *S. constricta*, indicating that the effects of Mg^2+^ on JSC growth likely involves the osmotic and ionic regulation caused by Mg^2+^ imbalance and its role as the activity center of metabolic and mineral enzymes. From the Mg^2+^ concentration distribution of the target-ISW, the higher Mg^2+^ concentration range 14.77–33.69 mmol⋅L^-1^ will significantly inhibit the shell length and body weight growth of JSC. This suggest that transplanting *S. constricta* in the target-ISW will not have growth inhibition caused by Mg^2+^ deficiency, and there may be only a response caused by excessive Mg^2+^.

Most evidences in bivalves is based on observations that on simulative conditions, oxygen consumption rapidly rises to levels above normal (Moon and Pritchard, 1970; Akberali et al., 1977; Almada-Villela, 1984). Both cell volume regulation and taurine efflux are inhibited in *Noetia ponderosa* erythrocytes and *Glycera dibranchiata* coelomocytes exposed to Ca^2+^-free hypoosmotic artificial seawater, and both are potentiated in high Ca^2+^ hypoosmotic artificial seawater (Smith and Pierce, 1987). Divalent cations also stabilize the cell membrane. The increased membrane permeability to taurine caused by Ca^2+^-free isosmotic media may be due to a lack of divalent cations that bind to and stabilize membranes (Swinehart et al., 1980). Not difficult to imagine the volume regulation of JSC haemocytes is extremely active, it may be the main reason sustained increase in the metabolism. Meanwhile, we found that the oxygen consumption increases within 24 h but it decreases sharply in 48 h. This suggest that high Ca^2+^ and Mg^2+^ stresses affects ventilatory activity causing internal hypoxia at 48 h. This may be the main reason for the rapid death of JSCs. On the contrary, Lee et al. (2005) noted that in Mg^2+^-free artificial seawater, the respiration rate of hard clams is decreased. No significant changes in oxygen consumption were observed in the DCMg-G1 group (Mg^2+^-free group), and it is likely that this inhibitory effect will take longer in JSCs.

When osmotic concentration and the ionic composition of the extracellular fluid be changed, the cells release cytoplasmic ions and organic osmolytes into the extracellular fluid to eliminate the osmotic gradient across the plasma membrane (Deaton, 2008; Larsen et al., 2014). Changes in environmental Ca^2+^ and Mg^2+^ have important regulation for both inorganic ion and organic osmotic regulation. In research of *G. dibranchiata*, it was found that the lack of Ca^2+^ ions in the environment did not lead to the efflux of Na^+^ and Cl^-^, but it caused the efflux of K^+^ (Costa and Pierce, 1983). Comparing our results, it suggesting that the effect of extracellular fluid Ca^2+^ concentration on NKA activity may be directly related to the flux of K^+^. We believe that the ionic regulation caused by Ca^2+^ may be much more than that. A decrease in the concentration of Mg^2+^ ions in water tends to inactivate NKA in shrimp (Roy et al., 2010). Similar results were also seen in our study, but Watts and Pierce (1978) reported that the ATPase in *Modiolus demissus* ventricles requires a minimum of 5 mM Mg^2+^ in body to maintain constant activity. These suggest that *S. constricta* may existence very high regulation ability. In addition, when cultured in Mg^2+^-free water, the NKA activity in JSC is not completely lost, as Mg^2+^ may be obtained through dietary Mg^2+^ ions in food (algae). Mollusc farming does not utilize compound feed directly, like shrimp farming, so the impact of dietary Mg^2+^ is difficult to eliminate. Of course, the target-ISW does not have the Mg^2+^-free type of water, and the target-ISW does not significantly inhibit the NKA activity when the Mg^2+^ content is the lowest. But the higher Mg^2+^ concentration range 14.77–29.54 mmol⋅L^-1^ may significantly increase the NKA activity of JSC. In addition, the Ca^2+^ concentration range of target-ISW may not significant impact on NKA activity of JSC. This suggest that when transplanting *S. constricta* in the target-ISW, Mg^2+^ may be one of the main factors to induce individuals to produce intense osmotic and ionic regulation.

Mg^2+^ operates synergistically to facilitate glutamate dehydrogenase activity and interactions between this glutamate dehydrogenase and aminotransferase (Fahien et al., 1985). In bivalve, inorganic ions are the first efflux components that respond to ambient medium cation ions (Na^+^, K^+^, Ca^2+^, and Mg^2+^) balance changes (Akberali et al., 1977; Shumway, 1977; Smith and Pierce, 1987). The efflux of inorganic ions induces the osmotic regulation from the organic components (free amino acids). The regulation of divalent cations in JSC may affect the concentration of free amino acids and may be directly related to changes in AST activity. Decreases and increases in Mg^2+^ concentration directly affect AST activity, and these results were recapitulated in our study. However, decreases and increases of Ca^2+^ concentration inhibit AST activity in JSC. So from the Ca^2+^ and Mg^2+^ concentration distribution of the target-ISW, we can imagine that both the higher Ca^2+^ (6.49–6.825 mmol⋅L^-1^) and Mg^2+^ (14.77–33.69 mmol⋅L^-1^) concentrations range may significantly affect AST activity. This indicating that changes of Ca^2+^ concentration in water not only affect digestive enzyme activity but also affect enzyme activity related to protein metabolism, thereby inhibiting growth (Wu et al., 2003).

The imbalance between Ca^2+^ and Mg^2+^ in water leads to increased oxygen consumption in aquatic animals, resulting in the production of more oxygen free radicals (Lin and Kao, 2000; Culotta, 2001). The increase in oxygen free radicals induces an increase in SOD activity to scavenge these radicals. In the present study, SOD activity is only augmented at moderately low calcium concentrations, which are coincident with high NKA activity and partial restoring of shell growth. SOD is also increased at the highest concentrations of both ions, which are very stressful conditions. But in target-ISW may be only the higher Mg^2+^ concentration range (14.77–33.69 mmol⋅L^-1^) may significantly increase the SOD activity of JSC. This suggest that Mg^2+^ may cause excessive metabolism and produce a large amount of oxygen free radicals when transplanting *S. constricta* in the target-ISW.

The AChE is a hydrolase for the signal termination at cholinergic synapses by rapid hydrolysis of the neurotransmitter acetylcholine (Quinn, 1987). AChE paly as a important role in neurotransmission process also in fish and invertebrates (Fulton and Key, 2001). The inhibition of AChE activity has been used widely as a biomarker of exposure to organotoxic substance and heavy metal ions in mollusk (Rickwood and Galloway, 2004; Devin et al., 2017). In addition, Pfeifer et al. (2005) reported that the activity of AChE in *Mytilus* sp. is sensitive to environmental salinity. Although the effect of the concentration of Ca^2+^ and Mg^2+^ on AChE activity in mollusc has not been clearly reported, in fish, Tomlinson et al. (1981) reported that AChE activity reached maximum saturation when both Ca^2+^ and Mg^2+^ concentrations were on the order of magnitude of 10 mmol, and the activity level were maintained at more higher Ca^2+^ and Mg^2+^ concentrations. The same situation be found in our study, AChE activity was continuously increased with increasing concentrations of Ca^2+^ and Mg^2+^ in water. Although there are some differences in the physiological functions of fish and bivalve, the physiological roles and functional mechanisms of AChE (the perspective of heavy metal ion binding) are relatively conservative. This indicating that the molecular mechanism of bivalve and fish AChE are similar, and the difference between the them may occur in the magnitude of the cation strength affecting the enzyme activity. In our study, indicating that the neurotransmission process of JSC be significant inhibition when the concentration of Ca^2+^ higher than 6.49 mmol L^-1^ and concentration of Mg^2+^ higher than 14.77 mmol L^-1^. Then the corresponding physiological processes of neuromodulation are inhibited. Obviously, this effect may be existed in both the higher Ca^2+^ (6.49–6.825 mmol⋅L^-1^) and Mg^2+^ concentration range (14.77–33.69 mmol⋅L^-1^) of target-ISW.

Lysozyme is a typical immunoenzyme that specifically lyses bacteria (Bachali et al., 2002; Xue et al., 2010). Usually LZM is divided into two categories, based on whether it can be combined with Ca^2+^ (Jielian et al., 2017). In mollusc, there is a class of LZM that binds to Ca^2+^ and interacts with calcium carbonate to participate in the shell formation process (Jimenez-Lopez et al., 2003). However, in mollusc is reported that LZM activity has a low tolerance to Ca^2+^ and Mg^2+^ concentrations (Nilsen et al., 1999; Olsen et al., 2003). In addition, the results of SOD and phagocytosis in this study also reflect the immune response of JSCs. So, we propose that the effects of Ca^2+^ and Mg^2+^ on the immune capacity of JSC are comprehensive. We suggest that (1) Ca^2+^ concentration (lower than 0.28 mmol L^-1^, higher than 19.46 mmol L^-1^) and Mg^2+^ concentration (higher than 14.77 mmol L^-1^) affect metabolic immune enzymes; (2) function of haemocyte phagocytosis will be inhibited when the concentration of Ca^2+^ and Mg^2+^ in hemolymph sharply rise or fall in short period of time. Because adhesion and aggregation function be limited (Chen and Bayne, 1995); (3) Ca^2+^ concentration (lower than 0.93 mmol L^-1^, higher than 6.49 mmol L^-1^) and Mg^2+^ concentration (lower than 0.37 mmol L^-1^, higher than 14.77 mmol L^-1^) affect antibacterial enzyme activity. Therefore, this means that when the *S. constricta* is transplanted to the target-ISW, both the higher Ca^2+^ (6.49–6.825 mmol⋅L^-1^) and Mg^2+^ (14.77–33.69 mmol⋅L^-1^) concentration range may significantly enhances the metabolic immunity, but the cellular immunity (haemocyte) and bacterial immunity are weakened.

The Ca^2+^/Mg^2+^ ratio is often reported in shrimp and has important effects on survival, growth and physiology (Pan et al., 2006; González-Vera and Brown, 2017). The Ca^2+^/Mg^2+^ ratio not only has strong species specificity, but also has been widely researched in shrimp species. Tavabe et al. (2013) found that a Ca^2+^/Mg^2+^ ratio of 0.8 was optimal for larval development, while Latif (1992) found that a 1:1 Ca^2+^/Mg^2+^ ratio was desirable for juveniles of *Macrobrachium rosenbergii*. This suggests that the Ca^2+^/Mg^2+^ ratio is an important indicator for shrimp, but further study is needed to elucidate its importance in other aquatic species. The Ca^2+^/Mg^2+^ ratio in mollusc has not been reported on. We do not have definitive evidence to obtain the significant correlation between Ca^2+^/Mg^2+^ ratio and the survival, growth, and physiology of JSC, but this does not mean that Ca^2+^/Mg^2+^ ratio has no effect, so this requires future validation with independent test.

Over the past 250 years, atmospheric carbon dioxide (CO_2_) levels increased by nearly 40% (Solomon et al., 2007). Ocean CO_2_ uptake, causes pH reductions and alterations in fundamental chemical balances that together are commonly referred to as ocean acidification (Millero et al., 2009). Orr et al. (2005) predicted that ocean acidification will cause a pH drop of 0.3–0.4 for the 21st century, is equivalent to approximately a 150% increase in H^+^ and 50% decrease in CO_3_^2-^ concentrations. Marine carbonates are mainly in the form of CaCO_3_ and MgCO_3_, which means that the concentration of Ca^2+^ and Mg^2+^ in the 21st century will increase within 50% than it is now. Comparing our findings, we can see that such an increase may not have a negative impact on the survival, growth and physiology of JSC. However, the negative impact of ocean acidification on the survival and growth of corals and molluscs is a proven fact (Hoegh-Guldberg et al., 2007; Fabry et al., 2008). Ocean acidification is a complex environmental change process involving not only changes in the concentration of metal cations, but also pH, *p*CO_2_, CO_3_^2-^, temperature and marine food chains (Fabry et al., 2008). Therefore, we believe that other factors or comprehensive factors other than Ca^2+^ and Mg^2+^ may eventually lead to the negative impact of ocean acidification on the survival and growth of molluscs.

## Conclusion

Stress caused by changes in the concentration of Ca^2+^ and Mg^2+^ in the environment may cause efflux and reflux of Ca^2+^ and Mg^2+^ in JSC. Metabolism and oxygen consumption are rapidly increased but haemocyte phagocytosis are decreased in short time. At this time, a large number of JSCs died when Ca^2+^ and Mg^2+^ exceed physiological limits. In the long-term stress, the imbalance both Ca^2+^ and Mg^2+^ may cause a comprehensive physiological response including ionic regulation, metabolic levels, neurotransmission process, and immunity. Then ultimately affect growth and even survival. When using such a “standard ruler” to measure the effect of Ca^2+^ and Mg^2+^ concentrations in the target-ISW on *S. constricta*, we found that only high concentrations of Ca^2+^ (3.24–6.825 mmol⋅L^-1^) and Mg^2+^ (14.77–33.69 mmol⋅L^-1^) had significantly impact on transplantation practice. In physiology, such effects include accelerated ionic regulation, accelerated metabolism, increased oxygen free radical production, increased metabolic immunity, decreased bacterial and cellular immunity, and inhibit neurotransmission process. The concentrations of Ca^2+^ and Mg^2+^ in the target-ISW does not cause all *S. constricta* to die in the short term, but it inhibits growth and causes a part of individual death during long-term aquaculture. Those implies that we have to choose the right concentration of Ca^2+^ and Mg^2+^ or to improve the water quality in target-ISW. Fortunately, the Ca^2+^ and Mg^2+^ content of most area target-ISWs is suitable for long-term transplantation of *S. constricta*. Our study also suggests that the increase in Ca^2+^ and Mg^2+^ ion concentrations caused by ocean acidification will not affect the survival, growth and physiology of *S. constricta*.

## Data Availability

All datasets generated for this study are included in the manuscript and/or the supplementary files.

## Ethics Statement

In this study, the animals were artificially propagated as juvenile clams.

## Author Contributions

MP was responsible for experimental design, test operations, data processing, and manuscript writing. ZL was responsible for data processing and manuscript modification. XL was responsible for experimental design. BY was responsible for test operations. TL was responsible for experimental data collection. JL, DN, and ZD were responsible for experimental program guidance.

## Conflict of Interest Statement

The authors declare that the research was conducted in the absence of any commercial or financial relationships that could be construed as a potential conflict of interest.
